# Hyperinsulinemia is a probable trigger for weight gain and hyperphagia in individuals with Prader‐Willi syndrome

**DOI:** 10.1002/osp4.663

**Published:** 2023-02-17

**Authors:** Frederick A. Kweh, Carlos R. Sulsona, Jennifer L. Miller, Daniel J. Driscoll

**Affiliations:** ^1^ Department of Pediatrics University of Florida College of Medicine Gainesville Florida USA; ^2^ Process and Analytical Development Resilience Biotechnologies, Inc. Alachua Florida USA; ^3^ Center for Epigenetics University of Florida College of Medicine Gainesville Florida USA

**Keywords:** hyperinsulinemia, hyperphagia, insulin, nutritional phase, obesity, Prader‐Willi

## Abstract

**Objective:**

Prader‐Willi syndrome (PWS) is the most frequently diagnosed genetic cause of early childhood obesity. Individuals with PWS typically progress through 7 different nutritional phases during their lifetime. The main objective of this study was to assess potential factors, particularly insulin, that may be responsible for the weight gains in sub‐phase 2a and their role in the subsequent increase in fat mass and obesity in sub‐phase 2b and insatiable appetite in phase 3.

**Methods:**

Fasting plasma insulin levels were measured in children with PWS between the ages of 0–12 years and in age‐matched non‐PWS participants with early‐onset major (clinically severe) obesity (EMO) and in healthy‐weight sibling controls (SC).

**Results:**

Participants with PWS in nutritional phases 1a and 1b had plasma insulin levels comparable to SC. However, the transition from phase 1b up to phase 3 in the PWS group was accompanied by significant increases in insulin, coinciding in weight gains, obesity, and hyperphagia. Only individuals with PWS in phase 3 had comparable insulin levels to the EMO group who were higher than the SC group at any age.

**Conclusions:**

Elevated insulin signaling is a probable trigger for weight gain and onset of hyperphagia in children with Prader‐Willi syndrome. Regulating insulin levels early in childhood before the onset of the early weight gain may be key in modulating the onset and severity of obesity and hyperphagia in individuals with PWS, as well as in other young children with non‐PWS early‐onset obesity. Preventing or reversing elevated insulin levels in PWS with pharmacological agents and/or through diet restrictions such as a combined low carbohydrate, low glycemic‐load diet may be a viable therapeutic strategy in combating obesity in children with PWS and others with early childhood obesity.

## Graphical abstract

Prader‐Willi syndrome (PWS) is the most frequently diagnosed genetic cause of early childhood obesity and individuals with PWS typically progress through 6 different postnatal nutritional phases during their lifetime. The results of our study suggest elevated insulin signaling may be a trigger for rapid weight gain early‐on in PWS. Our results also suggest hyperinsulinemia may promote leptin resistance, leading to development of obesity and hyperphagia later in childhood.

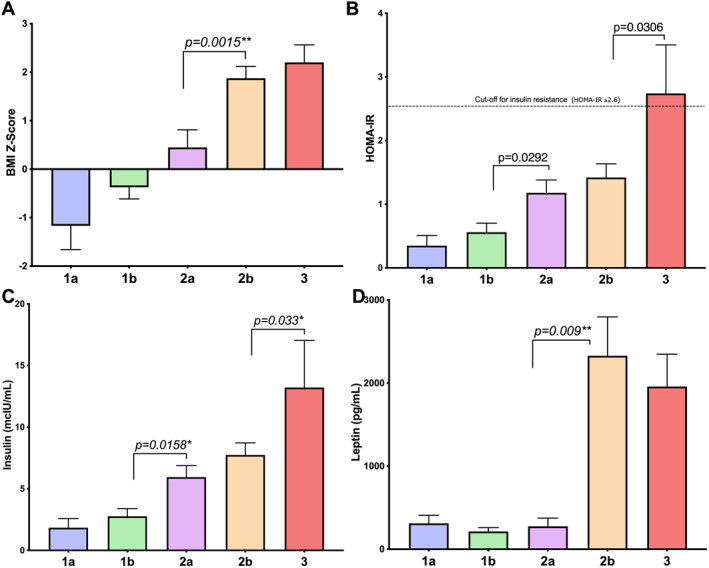

## 1 INTRODUCTION

Prader‐Willi syndrome (PWS) is a complex neurogenetic, endocrine and behavioral disorder caused by a lack of expression of paternally inherited imprinted genes in chromosomal region 15q11.2–q13 consequent to a paternal deletion of the region (60%–75% of individuals), maternal uniparental disomy 15 (25%–35%), or an imprinting defect (1%–4%).^1^, ^2^ PWS is the most frequently diagnosed genetic cause of early childhood obesity, occurring in approximately 1 in 10,000–25,000 live births.^1^, ^3^

In contrast to the long‐held view that there are two distinct nutritional phases in PWS (“failure to thrive” followed by “hyperphagia leading to obesity”), a 2011 longitudinal multicenter natural history study found that the transition between nutritional phases is much more complex, with one prenatal and 6 different postnatal nutritional phases through which individuals with PWS typically progress.^4^ Briefly, nutritional **Phase 0** occurs in utero with decreased fetal movements and growth restriction compared to unaffected siblings. In nutritional **Phase 1** the infant is hypotonic and without obesity, with sub‐phase **1a** characterized by poor appetite, hypotonia and difficulty feeding. This phase is followed by sub‐phase **1b** when the infant's appetite and feeding have significantly improved, and weight is increasing at a normal rate. Nutritional **Phase 2** is associated with an abnormal weight gain; in sub‐phase **2a** the weight increases without a significant change in appetite or caloric intake, while in sub‐phase **2b** the weight gain is associated with a concomitant increased interest in food, but the child can feel satiated. Nutritional **Phase 3** is characterized by an insatiable appetite typically accompanied by aggressive food‐seeking behavior and lack of satiety. Not all individuals with PWS go through all the stages described above, but the vast majority do. In addition, some adults progress to nutritional **Phase 4**, which is when an individual who was previously in **Phase 3** no longer has an insatiable appetite and is able to feel satiated.

The etiology behind the rapid weight gain in nutritional sub‐phase 2a without a significant increase in caloric intake remains unclear. This rapid increase in weight gain is further compounded by the subsequent development of hyperphagia and lack of satiety in sub‐phase 2b and nutritional phase 3, respectively.^4^, ^5^ The main objective of this study was to assess potential factors that may be behind the weight gains in sub‐phase 2a and their role in the subsequent increase in fat mass and obesity in sub‐phase 2b and insatiable appetite in phase 3. Because of its role in nutrient uptake, storage, and appetite regulation, the hypothesis was that increased insulin signaling may be a driving force behind nutritional sub‐phase 2a, sub‐phase 2b, and nutritional phase 3. Therefore, insulin was assessed as well as leptin, glucose, BMI z‐scores and homeostasis model assessment of insulin resistance (HOMA‐IR) levels in children with PWS with respect to nutritional phases relative to two control groups of similar age: healthy‐weight sibling controls (SC) and non‐PWS participants with early‐onset major (clinically severe) obesity (EMO) of unknown etiology. The SC and EMO groups provided appropriate controls for the PWS nutritional phases with respect to age, total fat mass, and BMI‐z scores.

## 2 METHODS

### 2.1 Participants

Forty participants with PWS between the ages of 5 weeks and 12 years were selected from a NIH funded PWS and EMO Natural History Study at the University of Florida for analysis of insulin levels in PWS in the current study. Individuals in this study were admitted for a 2‐day intensive phenotyping research study at the Clinical Research Center, Shands Hospital, University of Florida. Of the 40 participants with PWS, 20 had longitudinal studies with blood sampling at different nutritional phases during the course of the study. Nine of the 20 participants with longitudinal data also had 2 separate observations within the same nutritional phase. There were 2 individuals in phase 2a and 5 in phase 2b with repeated visits (2 observations). Phases 1a and 1b had 1 individual each with repeated visits (2 observations).

In addition, 39 non‐PWS participants with early‐onset major (clinically severe) obesity (EMO) of unknown etiology and 52 healthy‐weight control siblings (SC) of PWS or EMO participants between the ages of 5 weeks to 12 years and of similar weight and sex were also selected to serve as comparison groups for this study. However, after screening for individuals on medications that could potentially impact insulin levels, only 24 of the 39 EMO participants were included in this study. Each study group was composed of participants from different racial/ethnic backgrounds consisting of European Americans (35 PWS, 45 SC, and 15 EMO), African Americans (3 PWS, 3 SC, and 3 EMO), Asian Americans (1 EMO) and Hispanic Americans (2 PWS, 4 SC, and 5 EMO). Characteristics of the three groups are shown in Table 1 for ages 0 < 13 years. Participants were limited to <13 years of age to minimize the effects of puberty. The majority (75%) of the participants were in pubertal stage 1 and none were in stage 5 (Table S1). Appropriate genetic testing was used to classify individuals with PWS into the appropriate molecular class—deletion (del), uniparental disomy (UPD) or an imprinting defect (ID).^1^ Participants on medications that may affect insulin levels (such as insulin or metformin) were excluded from this study.

**TABLE 1:** Characteristics of study participants by age groups.

0 < 2 years	Phase 1a	Phase 1b	SibC	EMO
Participants	8 (2F, 6M)	12 (6F, 6M)	9 (5F, 4M)	N/A
EA/AA/HA/AsA	5/2/1/0	10/0/2/0	8/1/0/0	N/A
Mean age (years)	0.72 ± 0.39	1.53 ± 0.51	1.05 ± 0.71	N/A
Mol.Class (Del/UPD/ID)	4/3/1	6/5/1	N/A	N/A
GHT %	38%	75%	N/A	N/A
Mean GHT age (years)	1.11	1.62	N/A	N/A

2 < 5 years	Phase 2a	Phase 2b	SibC	EMO
Participants	17 (10F, 7M)	13 (4F, 9M)	18 (12F, 6M)	8 (1F, 7M)
EA/AA/HA/AsA	17/0/0/0	10/2/1/0	14/1/3/0	5/1/2/0
Mean age (years)	3.68 ± 0.92	3.46 ± 0.78	3.81 ± 0.84	4.11 ± 0.87
Mol.Class (Del/UPD/ID)	9/6/2	10/3/0	N/A	N/A
GHT %	100%	85%	N/A	N/A
Mean GHT age (years)	3.68	3.54	N/A	N/A

5 < 13 years	Phase 2b	Phase 3	SibC	EMO
Participants	12 (6F, 6M)	10 (3F, 7M)	45 (29F, 16M)	18 (11F, 7M)
EA/AA/HA/AsA	12/0/0/0	9/0/1/0	44/1/0/0	11/2/4/1
Mean age (years)	7.90 ± 1.76	8.54 ± 3.31	7.87 ± 2.15	8.50 ± 2.39
Mol.Class (Del/UPD/ID)	10/2/0	6/4/0	N/A	N/A
GHT %	75%	80%	N/A	N/A
Mean GHT age (years)	7.39	7.78	N/A	N/A

*Note*: Age expressed as Mean ± standard deviation.

Abbreviations: AA, African American; AsA, Asian American; Del, Paternal Deletion; EA, European American; F, Female; GHT %, Percent of PWS Individuals on Growth Hormone Therapy; HA, Hispanic American; ID, Imprinting Defect; M, Male; Mol.Class, Molecular Class; N/A, Not Applicable; UPD, Maternal Uniparental Disomy 15.

The individuals in the EMO group are a rather remarkable group who were diagnosed with obesity (>97th percentile) on the Centers for Disease Control (CDC) body mass index (BMI) curve (www.cdc.gov) before 4 years of age, remained this way, and were typically hyperphagic. All participants with EMO included in this study had a normal chromosomal microarray (CMA), normal DNA methylation analysis for PWS, detectable serum leptin, and absence of a melanocortin‐4 receptor (MC4R) deficiency by mutational analysis. BMI z‐scores were calculated from body weight and height measurements using the United States Centers for Disease Control (CDC) criteria. The CDC recommends using World Health Organization (WHO) weight‐for‐length curves for children less than 2 years in age rather than BMI (CDC Centers for Disease Control and Prevention). Body composition was determined by dual‐energy x‐ray absorptiometry (DEXA). The data were analyzed with PC PAL Growth XP v1.0 software and the total body fat was expressed as a percentage of body weight.

### 2.2 Assessment of nutritional phases in PWS

Two physician scientists with significant expertize in PWS (DJD and JLM) reviewed the characteristics of the nutritional phases with the families of children with PWS based on the criteria delineated in the original manuscript describing the nutritional phases of PWS.^4^ After independently assessing the nutritional phase of the individual, the investigators conferred and came to a decision regarding what nutritional phase each patient was in at the time of evaluation. Any discrepant decisions were reviewed in order to come to a unanimous decision on the nutritional phase.

### 2.3 Measurement of insulin, glucose, and leptin

Blood samples were obtained from participants as previously described.^6^ Briefly, children over the age of 1 year underwent a 12 h overnight fast, while those less than 1 year of age had an 8 h overnight fast. Blood was collected between 08:00 AM and 09:00 AM, after an overnight fast and again at 1 h and 2 h post oral glucose tolerance test (OGTT), in serum separator tubes containing 100 μL aprotinin (~10,000 KIU/mL) and allowed to sit at room temperature for 15–30 min until clotting, while plasma blood samples were collected in EDTA blood collection tubes with 100 μL aprotinin, and then centrifuged at 3000 rpm (1800 × g) for 10 min at +4°C. All samples were stored at −80°C until use.

Blood insulin was measured on a Beckman‐Coulter Dxl 800 analyzer using the Access Ultrasensitive Insulin Assay. The method was a non‐competitive immunoassay with chemiluminescent detection. The analytical %CV ranged from 5.0% to 8.5% and the limit of detection was 0.03 µIU/mL. Blood glucose was also measured on the Beckman‐Coulter Dxl 800 analyzer using standardized kits. Briefly, glucose is phosphorylated by hexokinase (HK) in the presence of adenosine triphosphate (ATP) and magnesium ions to produce glucose‐6‐phosphate (G‐6‐P) and adenosine diphosphate (ADP). Glucose‐6‐phosphate dehydrogenase (G6P‐DH) specifically oxidizes G‐6‐P to 6‐phosphogluconate with the concurrent reduction of nicotinamide adenine dinucleotide (NAD+) to nicotinamide adenine dinucleotide, reduced (NADH). The change in absorbance at 340/660 nm is proportional to the amount of glucose present in the sample (Package Insert: OSR General Chemistry BAOSR6x21.04, 20143‐04). The insulin and glucose data were used to calculate the HOMA‐IR value for each individual at the different nutritional phases or age. Plasma leptin and C‐peptide were measured in duplicates using the metabolic panel of the Luminex assay system per instructions of the manufacturer (Millipore Inc, CA, USA). The limit of detection for the Luminex human leptin and C‐Peptide assays were 64 pg/mL and 16 pg/mL, respectively. The intra‐ and inter‐assay variabilities of the assays were 5%–10% and <15% respectively. The assay data was normalized for inter‐assay variability to four internal controls that were included with every assay performed.

### 2.4 Statistical analysis

For assessment of fasting insulin, glucose and leptin levels, only participants with an accompanying fasting C‐peptide value were included in the analyses. While C‐peptide is not needed for the determination of insulin resistance by HOMA‐IR, the C‐peptide data was used to confirm that differences in insulin levels observed between the PWS and SC groups were not the consequence of differences in whole body insulin clearance.^7^ Two sets of analyses were conducted using the JMP statistical software package (JMP, Cary, NC, USA). The first set of analyses looked at whether insulin levels differ significantly between the PWS nutritional phases and whether nutritional phase insulin levels correlated with changes in BMI z‐scores within the phases. The second set of analyses contrasted PWS nutritional phase insulin, glucose, HOMA‐IR and BMI z‐scores relative to normal healthy‐weight sibling controls (SC) of similar age, and other non‐PWS individuals with early‐onset major (clinically severe) obesity (EMO).

#### 2.4.1 Analysis of nutritional phase

A mixed model analysis with participants as random variables to account for repeated measures, insulin as dependent variable, and nutritional phase as independent variable was used to assess PWS insulin levels. This was to control the study‐wide false discovery rate by testing the null hypothesis that there was no difference in nutritional phase insulin levels. Had that been non‐significant (*p* > 0.05), the analysis would have been terminated. Furthermore, bivariate analysis was used to examine the relationship between insulin and BMI z‐scores by nutritional phases.

#### 2.4.2 Comparisons with controls

Classification of the six postnatal nutritional phases in PWS have previously been described by Miller and colleagues.^4^ PWS nutritional phase insulin levels were compared to normal‐weight sibling controls (SC) and non‐PWS individuals with early‐onset major (clinically severe) obesity (EMO) of similar age as follows: Phase 1a and 1b versus 0 < 2 years SC; Phase 2a and 2b versus 2 < 5 years SC/EMO; Phase 2b and Phase 3 versus 5 < 13 years SC/EMO. Personal averages (Mean of Means) were taken within each group and contrasted in an analysis of variance. This analysis is aimed at making the inference at the participant level, equalizing the weight of each contributing participant regardless of how many observations the participant contributes within the age group. Comparisons between the SC and EMO control groups allowed an examination of the differences between lean and non‐PWS populations with obesity of similar age and sex.

This study was approved by the University of Florida Institutional Review Board and all adult participants or guardians provided written informed consent and, where appropriate, participants provided assent.

## 3 RESULTS

### 3.1 Insulin

**FIGURE 1:**
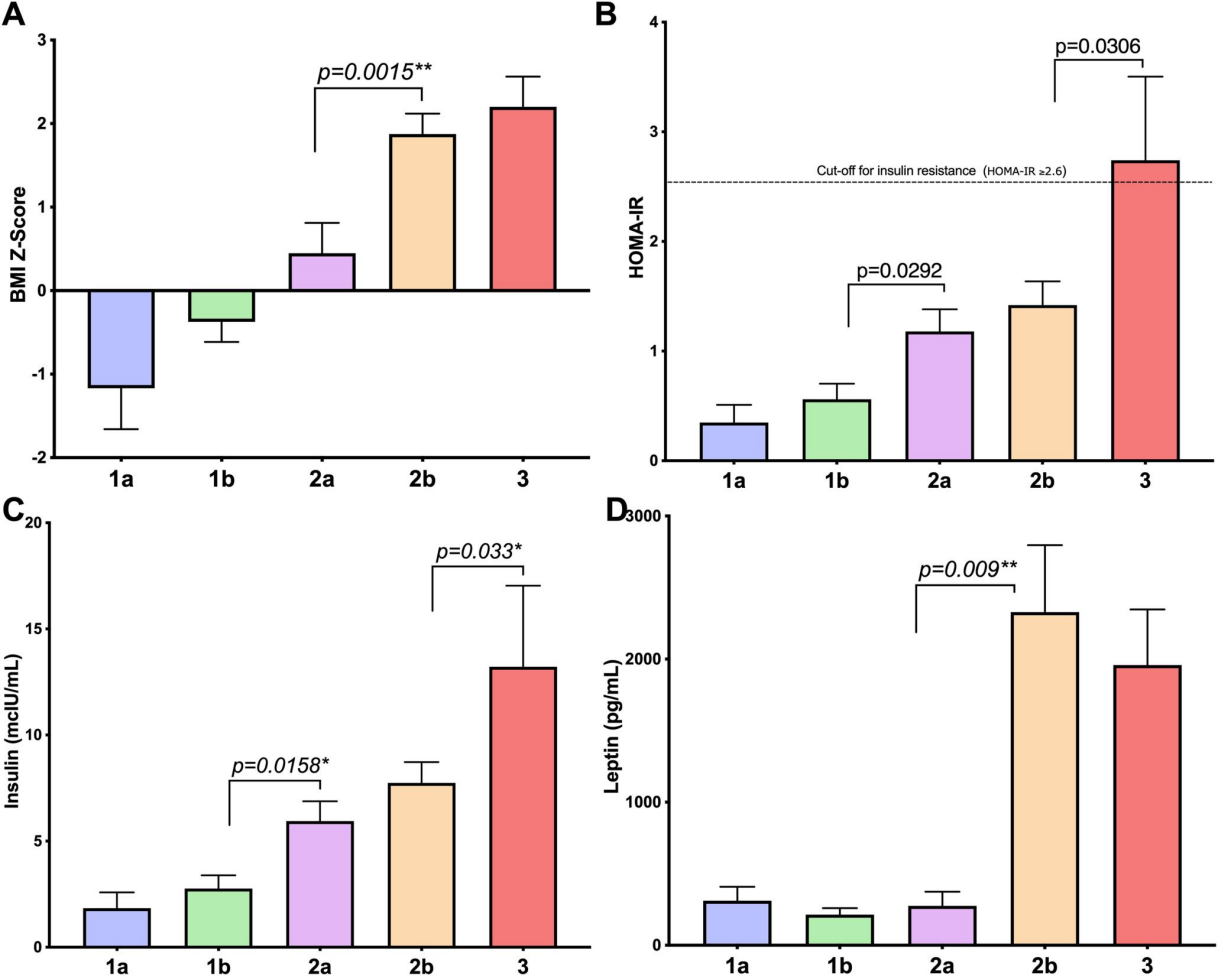
Analysis of changes in Prader‐Willi syndrome nutritional phase: (A) body mass index (BMI) z‐scores—note significant difference between 2a and 2b; (B) homeostasis model assessment of insulin resistance—values above 2.6 represent insulin resistance; (C) plasma insulin—note significant increase from 1b to 2a and from 2b to 3; (D) plasma leptin levels—note significant increase from 2a to 2b, coinciding with increase in BMI‐z. N.B. Graphs represent mean and standard error of the mean.

**FIGURE 2:**
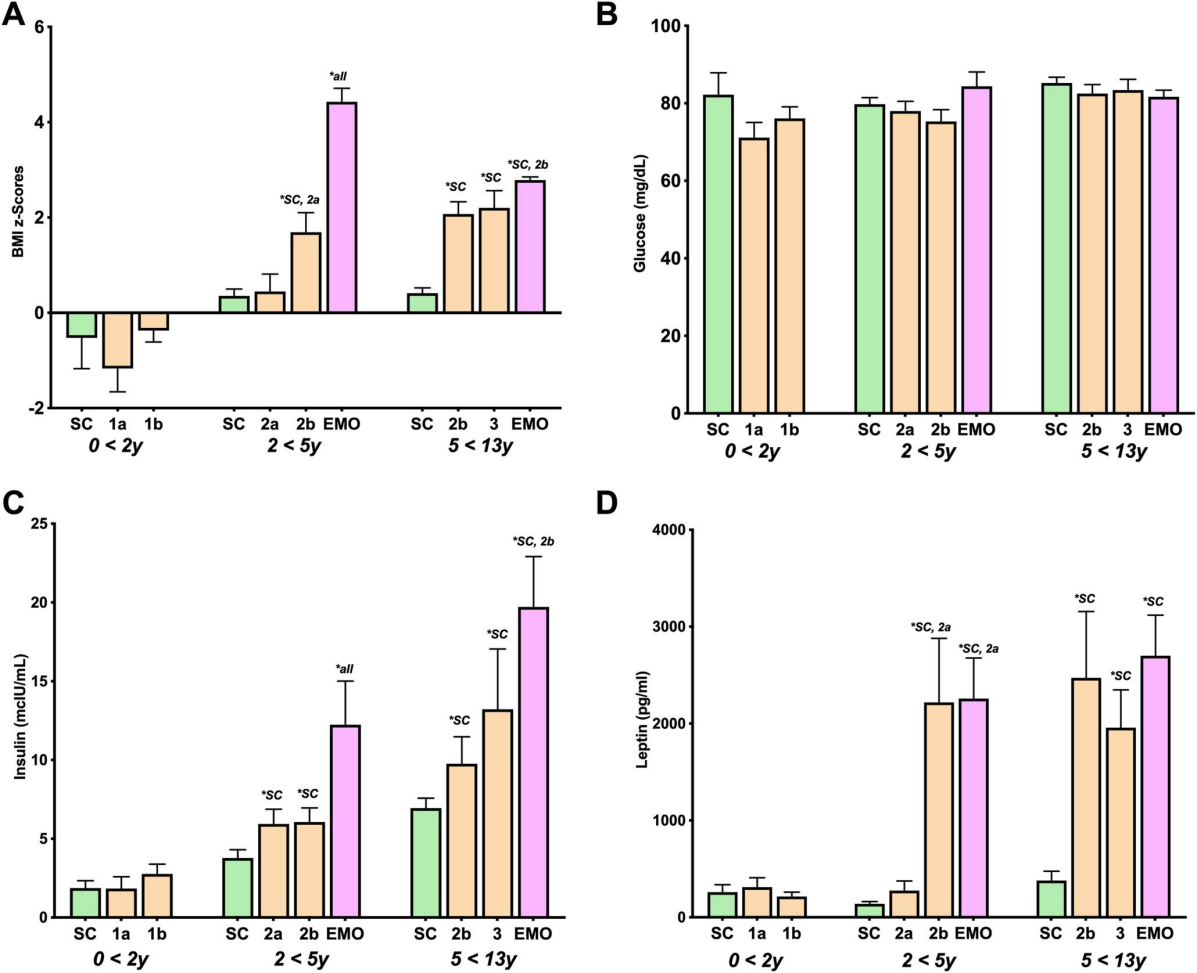
Group comparative analysis of individuals with Prader‐Willi syndrome at different nutritional phases relative to healthy‐weight sibling controls (SC) and others with early‐onset major (clinically severe) obesity (EMO) of similar age: (A) BMI z‐scores, (B) plasma glucose, (C) plasma insulin, (D) plasma leptin. * represents significant difference (*p < 0.05*) of current group with indicated comparison group(s).

Fasting plasma insulin levels did not change significantly with transition from nutritional phase 1a to 1b or from phase 2a to 2b (Table 2). However, the transition from phase 1b to phase 2a (mean, standard error, *p* value: 2.8 ± 0.6 vs. 5.9 ± 0.9; *p* = 0.016), and from phase 2b to phase 3 (6.9 ± 1.0 vs. 13.2 ± 3.8; *p* < 0.033) were associated with significant increases in fasting plasma insulin levels (Figure 1C; Table 2). When nutritional phase insulin levels were assessed relative to healthy‐weight sibling controls (SC) and EMO controls of similar age, PWS individuals in nutritional phase 1a and 1b had normal fasting plasma insulin levels, while individuals in phase 2a (5.9 ± 0.9 vs. 3.8 ± 0.5; *p* = 0.017), 2b (6.1 ± 0.9 vs. 3.8 ± 0.5; *p* = 0.019) and 3 (13.2 ± 3.8 vs. 6.9 ± 0.6; *p* < 0.001) had significantly elevated fasting insulin levels relative to the sibling controls, but were lower than the EMO group (Figure 2C; Tables 3 and 4).

**TABLE 2:** PWS Nutritional phase clinical and hormonal characteristics.

Variable	Phase 1a	Phase 1b	Phase 2a	Phase 2b	Phase 3	*p‐Values*
Sample size	8	12	17	25	10	
Sex	2F, 6M	6F, 6M	10F, 7M	10F, 15M	3F, 7M	
Del/UPD/ID	4/3/1	6/5/1	9/6/2	20/5/0	6/4/0	
Age range (years)	0.11–1.26	0.18–1.99	2.10–4.80	1.95–10.44	3.93–12.78	
Mean age (years)	0.73(0.14)[8]	1.53(0.14)[12]	3.67(0.22)[17]	5.59(0.52)[25]	8.54(1.05)[10]	
BMI Z‐score	−1.17(0.49)[8]	−0.37(0.24)[12]	0.45(0.36)[17]	1.91(0.25)[24]	2.20(0.36)[10]	^b^**
DEXA	22.4(4.1)[8]	19.6(2.1)[12]	22.4(3.1)[17]	36.6(2.7)[22]	40.1(4.3)[7]	^b^*
Insulin (mcIU)	1.8(0.7)[8]	2.8(0.6)[12]	5.9(0.9)[17]	6.9(1.0)[25]	13.2(3.8)[10]	^a^*, ^c^*
C‐peptide (pmol/L)	113(25)[8]	163(19)[12]	235(28)[17]	307(46)[25]	447(88)[10]	^a^*, ^c^*
Glucose (mg/dL)	71(4)[8]	76 (3)[12]	78 (2)[17]	79(2)[25]	83(3)[10]	
HOMA‐IR	0.35(1.60)[8]	0.56(0.14)[12]	1.18(0.20)[17]	1.42(0.22)[25]	2.74(0.76)[10]	^a^*, ^c^*
Leptin (pg/mL)	311(97)[8]	214(45)[12]	613(265)[17]	2328(467)[23]	1958(389)[10]	^b^**

*Note*: Data expressed as mean with standard error (SE) and sample size [n]; N/A, Not Applicable; F, Female; M, Male. For 0–2 years, WHO weight‐for‐length z‐Scores used; After 2 years CDC BMI z‐Scores used; *p‐*values less than 0.05 are considered significant.

Abbreviations: Del, Deletion; F, Female; HOMA‐IR, homeostasis model assessment of insulin resistance; ID, Imprinting Defect; M, Male; N/A, Not Applicable; UPD, Uniparental Disomy.

^a^ Phase 1b versus Phase 2a.

^b^ Phase 2a versus Phase 2b.

^c^ Phase 2b versus Phase 3.

*p < 0.05, **p < 0.01.

**TABLE 3:** PWS Nutritional phase clinical and hormonal characteristics relative to lean controls and controls with obesity of similar age.

0 < 2 years	Phase 1a	Phase 1b	SibC	EMO
Sample size and sex	8 (2F, 6M)	12 (6F, 6M)	9 (5F, 4M)	
Mean age (years)	0.72 ± 0.39	1.53 ± 0.51	1.05 ± 0.71	N/A
Insulin (mcIU)	1.8 ± 0.7	2.8 ± 0.6	1.9 ± 2.2	N/A
Glucose (mg/dl)	71 ± 4	76 ± 3	82 ± 6	N/A
Leptin (pg/mL)	311 ± 97	214 ± 45	259 ± 77	N/A
HOMA‐IR	0.35 ± 0.16	0.56 ± 0.14	0.40 ± 0.11	N/A
BMI z‐score	−1.17 ± 0.49	−0.37 ± 0.24	−0.53 ± 0.65	N/A
% Body fat by DEXA	22.4 ± 4.0	19.6 ± 2.2	17.3 ± 1.4	N/A

2 < 5 years	Phase 2a	Phase 2b	SibC	EMO
Sample size and sex	17 (10F, 7M)	13 (4F, 9M)	18 (12F, 6M)	8 (1F, 7M)
Mean age (years)	3.68 ± 0.92	3.46 ± 0.78	3.81 ± 0.84	4.11 ± 0.87
Insulin (mcIU)	5.9 ± 0.9	6.1 ± 0.9	3.8 ± 0.5	12.2 ± 2.8
Glucose (mg/dl)	78 ± 2	75 ± 3	80 ± 2	84 ± 4
Leptin (pg/mL)	651 ± 279	2218 ± 660	139 ± 23	2258 ± 418
HOMA‐IR	1.18 ± 0.20	1.12 ± 0.20	0.75 ± 0.11	2.63 ± 0.63
BMI z‐score	0.45 ± 0.36	1.69 ± 0.41	0.35 ± 0.14	4.43 ± 0.28
% Body fat by DEXA	22.4 ± 3.1	29.0 ± 2.8	18.2 ± 1.5	44.6 ± 2.3

5 < 13 years	Phase 2b	Phase 3	SibC	EMO
Sample size and sex	12 (6F, 6M)	10 (3F, 7M)	45 (29F, 16M)	18 (11F, 7M)
Mean age (years)	7.90 ± 1.76	8.54 ± 3.31	7.87 ± 2.15	8.50 ± 2.39
Insulin (mcIU)	9.8 ± 1.7	13.2 ± 3.8	6.9 ± 0.6	19.7 ± 3.2
Glucose (mg/dl)	82 ± 2	83 ± 3	85 ± 2	82 ± 2
Leptin (pg/mL)	2471 ± 684	1958 ± 389	379 ± 96	2700 ± 418
HOMA‐IR	1.74 ± 0.38	2.74 ± 0.76	1.50 ± 0.15	3.91 ± 0.61
BMI z‐score	2.07 ± 0.26	2.20 ± 0.36	0.41 ± 0.11	2.79 ± 0.06
% Body fat by DEXA	44.3 ± 3.2	40.1 ± 4.3	19.9 ± 1.0	46.8 ± 1.2

*Note*: All data expressed as Mean ± SE (Standard Error of the mean).

Abbreviations: HOMA‐IR, homeostasis model assessment of insulin resistance; N/A, Not Applicable.

**TABLE 4:** *p*‐values of PWS nutritional phase comparisons to normal weight controls and controls with obesity.

0 < 2 years	1a versus 1b	1a versus SibC	1b versus SibC
Insulin (mcIU)	0.2921	0.5140	0.7286
Glucose (mg/dl)	0.2725	0.0967	0.3689
Leptin (pg/mL)	0.2640	0.4933	0.5982
HOMA‐IR	0.2874	0.4431	0.7612
BMI z‐score	0.2182	0.0956	0.2123
DEXA	0.5196	0.2654	0.4478

2 < 5 years	2a versus 2b	2a versus SibC	2a versus EMO	2b versus SibC	2b versus EMO	SibC versus EMO
Insulin (mcIU)	0.8930	0.0171*^a^	0.0255*	0.0196*	0.0345*	0.0003***
Glucose (mg/dl)	0.6542	0.7195	0.2376	0.3615	0.1264	0.2361
Leptin (pg/mL)	0.0093**	0.2900	<0.0001****	0.0017**	0.8579	<0.0001****
HOMA‐IR	0.9001	0.0273*	0.0212*	0.0302*	0.0309*	0.0004***
BMI z‐score	0.0314*	0.9747	<0.0001****	0.0057**	0.0001***	<0.0001****
DEXA	0.1408	0.2026	<0.0001****	0.0009***	0.0008***	<0.0001****

5 < 13 years	2b versus 3	2b versus SibC	2b versus EMO	3 versus SibC	3 versus EMO	SibC versus EMO
Insulin (mcIU)	0.2448	0.0057**	0.0547	0.0002****	0.5386	<0.0001****
Glucose (mg/dl)	0.3703	0.6819	0.9918	0.6207	0.2747	0.5487
Leptin (pg/mL)	0.2135	<0.0001****	0.8728	<0.0001****	0.1354	<0.0001****
HOMA‐IR	0.2261	0.0096**	0.0666	0.0001***	0.6222	<0.0001****
BMI z‐score	0.7715	<0.0001****	0.0062**	<0.0001****	0.0674	<0.0001****
DEXA	0.4406	<0.0001****	0.4211	<0.0001****	0.0590	<0.0001****

Abbreviation: HOMA‐IR, homeostasis model assessment of insulin resistance.

^a^ Onset of increased insulin.

*p < 0.05, **p < 0.01, ***p < 0.001, ****p < 0.0001.

### 3.2 Glucose

Fasting glucose levels did not change significantly with transition through the nutritional phases (Table 2). When nutritional phase glucose levels were assessed relative to SC and EMO groups of similar age, no significant differences were observed between PWS fasting plasma glucose levels and SC or EMO individuals at any nutritional phase (Figure 2B; Tables 3 and 4).

### 3.3 HOMA‐IR

There was a general upward trend in HOMA‐IR values with transition through the nutritional phases (Table 2; Figure 1B) however, participants with PWS in nutritional phases 1a, 1b, 2a, and 2b did not reach the cut‐off point (HOMA‐IR of 2.6) of what is considered to be insulin resistance.^8^, ^9^ When nutritional phase HOMA‐IR values were assessed relative to the SC and EMO group, the PWS groups in nutritional phase 1a and 1b had normal HOMA‐IR values relative to SC, while individuals in phase 2a (1.18 ± 0.20 vs. 0.75 ± 0.11; *p* = 0.027) and 2b (1.12 ± 0.20 vs. 0.75 ± 0.11; *p* = 0.300) had significantly elevated HOMA‐IR values relative to SC, but significantly lower (1.18 ± 0.20 vs. 2.63 ± 0.63; *p* = 0.021 and 1.12 ± 0.20 vs. 2.63 ± 0.63; *p* = 0.030) relative to the EMO group (Tables 3 and 4). Only PWS individuals in nutritional phase 3 had HOMA‐IR values similar to the EMO group and above the cut‐off point of 2.6 for insulin resistance (Table 3). HOMA‐IR values for the EMO group were significantly elevated relative to the SC group at any age, and above the cut‐off point for insulin resistance (Tables 3 and 4). There was no significant impact of growth hormone therapy (GHT) found on insulin/HOMA‐IR values within nutritional phases in PWS when comparing those on GHT versus those who were not on GHT, however this may be due to the relatively small number of participants with PWS who were not on GHT.

### 3.4 Leptin

Fasting plasma leptin levels were similar among PWS patients in nutritional phase 1a, 1b and 2a, however, the transition from phase 2a to phase 2b was associated with a significant increase in plasma leptin (613 ± 265 vs. 2328 ± 467; *p* = 0.009) (Figure 1D; Table 2). When nutritional phase leptin levels were assessed relative to healthy‐weight sibling controls (SC) of similar age, PWS individuals in phase 1a, 1b and 2a had similar leptin levels to the SC group, while individuals in phase 2b (2471 ± 684 vs. 379 ± 96; *p* < 0.001) and 3 (1958 ± 389 vs. 379 ± 96; *p* < 0.001) had significantly elevated plasma leptin levels compared to the SC group, and were comparable to the EMO group (Figure 2D; Tables 3 and 4).

### 3.5 BMI z‐scores and body composition

Infants with PWS in phase 1a and 1b had similar BMI z‐scores. However, the transition from phase 2a to 2b was associated with a significant increase in BMI z‐scores (0.45 ± 0.36–1.91 ± 0.25, *p* = 0.002) (Figure 1A; Table 2). Surprisingly, the weight gain in phase 2a was not associated with significant changes in total body fat (DEXA), while the weight gain in phase 2b was accompanied by a significant increase in total body fat (2a vs. 2b: 22.40 ± 3.1 vs. 36.60 ± 2.7, *p* < 0.050; Table 2). Infants with PWS in phase 1a had more total body fat relative to the SC group despite having BMIs that were on average 15% lower than SC (Figure 2A, Tables 3 and 4) as previously reported.^4^

### 3.6 Oral glucose tolerance test (OGTT)

**FIGURE 3:**
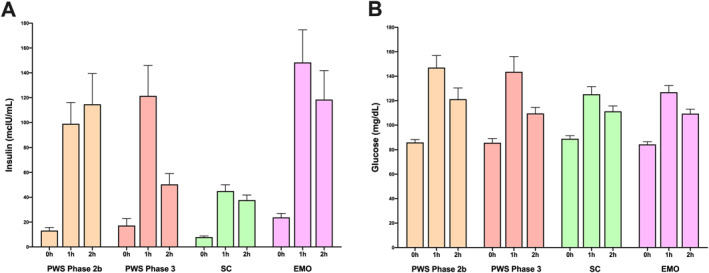
Oral glucose tolerance test (OGTT) of individuals with Prader‐Willi syndrome at nutritional phases 2b and 3, healthy‐weight sibling controls (SC), and others with early‐onset major (clinically severe) obesity (EMO) of similar age: (A) plasma insulin levels at baseline, 1 and 2h post OGTT; (B) plasma glucose levels at baseline, 1 and 2h post OGTT.

An oral glucose tolerance test (OGTT) was administered for post‐glucose load assessment and comparative analysis of insulin sensitivity and glucose tolerance between hyperphagic participants with PWS in nutritional phase 2b and phase 3 and age‐matched normal‐weight sibling controls, as well as other hyperphagic individuals with EMO The OGTT data shows an extended second‐phase insulin secretory response post glucose challenge among PWS individuals in phases 2b and 3, as well as in age‐matched EMO participants, relative to normal‐weight sibling controls even though there was no significant difference in blood glucose levels among the groups (Figure 3A,B; Table 5).

**TABLE 5:** Oral glucose tolerance test.

5 < 13 years	PWS—Phase 2b			PWS—Phase 3			SC			EMO		
OGTT	0h	1h	2h	0h	1h	2h	0h	1h	2h	0h	1h	2h
Sample size	15	15	15	11	11	11	20	20	20	26	26	26
Sex	10F/5M			7F/4M			13F/7M			12F/14M		
Insulin (mcIU)	13 ± 2	99 ± 17*	114 ± 25*	17 ± 6	121 ± 24*	50 ± 9	8 ± 1	45 ± 5	38 ± 4	24 ± 3	148 ± 26*	118 ± 23*
Glucose (mg/dl)	86 ± 2	147 ± 10	121 ± 9	86 ± 3	143 ± 12	109 ± 5	89 ± 2	125 ± 6	111 ± 4	84 ± 2	127 ± 5	109 ± 4
HOMA‐IR	2.94 ± 0.62			4.01 ± 1.54*			1.80 ± 0.23			4.95 ± 0.66*		
BMI z‐score	1.86 ± 0.22*			2.20 ± 0.25*			0.54 ± 0.15			2.51 ± 0.13*		

*Note*: All data expressed as Mean ± SE. Not all participants with OGTT had values for C‐peptide at baseline.

Abbreviation: HOMA‐IR, homeostasis model assessment of insulin resistance.

*p < 0.05 relative to SC (normal‐weight sibling controls).

## 4 DISCUSSION

Insulin plays an important role in weight gain as it facilitates the uptake of glucose and free fatty acids in adipocytes for conversion to triglycerides while inhibiting lipolysis, resulting in increased fat storage.^10^, ^11^, ^12^, ^13^, ^14^, ^15^ Fasting hyperinsulinemia is a major predictor of weight gain in children^16^, ^17^ while inhibition of insulin secretion has been reported to promote weight loss.^11^, ^18^ In the current study, significant increases in plasma insulin levels were observed for PWS individuals transitioning into nutritional phase 2a and phase 3.

Individuals with PWS have been reported to have less insulin resistance relative to other populations with obesity.^19^ The data show that the transition from phase 1b into phase 2a was marked by weight gain and a significant increase in fasting plasma insulin levels and HOMA‐IR, but without significant changes in total fat mass (DEXA). The increase in fasting plasma insulin levels and HOMA‐IR are suggestive of developing disruption in hepatic insulin action.^20^

Insulin has been reported to promote glucose uptake in the muscles and physiological hyperinsulinemia has been shown to promote muscle protein synthesis and muscle anabolism.^21^ It is possible that the initial weight gain in nutritional phase 2a results from impaired central and hepatic insulin signaling driving increased peripheral insulin secretion and a general increase in lean mass, while the onset of obesity in phase 2b is driven primarily by increased total fat mass and leptin due to elevated insulin signaling in adipose tissue.^22^, ^23^ The data support this postulation, showing an increase in body mass without a significant increase in fat mass occurring in phase 2a,while a significant increase in BMI z‐score, leptin and fat mass (by DEXA) was observed in individuals transitioning from phase 2a to phase 2b.

Interestingly, the rise in plasma insulin noted appears to coincide with changes observed in the acylated (AG) and unacylated ghrelin (UAG) concentrations in children with PWS when transitioning through the nutritional phases.^24^ Whether or not ghrelin dysregulation promotes hyperinsulinemia in PWS is beyond the scope of this manuscript and requires further investigation.

The transition to nutritional phase 2b coincided with a significant increase in leptin levels in PWS. Leptin plays a critical role in the regulation of appetite and satiety^25^, ^26^ and elevated leptin levels have been demonstrated to correlate with hyperphagia and leptin resistance in other populations with obesity.^27^ Nutritional phase 2b is characterized by the onset of obesity and hyperphagia in PWS. Given that insulin is known to interfere with leptin signaling in the hypothalamus and promote the development of central leptin resistance, increased appetite, and a greater fat mass,^11^, ^14^, ^28^ the proposed hypothesis is that peripheral and central hyperinsulinemia in nutritional phase 2a triggers leptin resistance centrally and peripherally. Furthermore, hyperinsulinemia might facilitate the development of central insulin and leptin resistance and be the underlying cause for the onset of hyperphagia in nutritional phase 2b and lack of satiety in phase 3.

Central and peripheral insulin signaling are both necessary for normal nutrient handling and disruption of either signaling pathway results in hyperinsulinemia, hyperphagia and obesity.^20^, ^22^, ^29^ This study reports the presence of hyperinsulinemia in individuals with PWS. The data suggest elevated insulin signaling (hyperinsulinemia) promotes weight gain and an increase in fat mass with a concomitant increase in circulating leptin levels inducing central insulin and leptin resistance, subsequently leading to obesity, hyperphagia and lack of satiety. Hypothalamic dysfunction combined with decreased insulin receptors and altered mitochondrial bioenergetics in individuals with PWS^30^, ^31^, ^32^ may underlie the early increases in insulin secretion and subsequent hyperinsulinemia.

It is possible that nutritional phases 0 and 1a of PWS contribute to the insulin pathology seen in this study. Nutritional phase 0, which occurs in utero, is associated with a smaller birthweight and phase 1a is associated with a “failure to thrive” phenotype. Holland and colleagues have proposed that aspects of the PWS phenotype are consequent of downregulation of placental‐fetal nutritional pathways which result in “relative fetal starvation”.^33^ Thus, it is possible that these infants are set up for insulin hypersecretion similar to the “thrifty phenotype” hypothesis.^34^ This is compounded by the development of hypothalamic insulin resistance, which sets up a situation of unchecked caloric intake in the face of positive energy balance. Furthermore, hypothalamic insulin resistance interferes with meal‐associated insulin effects on dopamine reuptake and the extinguishing of the hedonic system at the nucleus accumbens, causing a derangement of homeostatic feeding and promoting increased energy intake by way of reward‐induced (hedonic) overeating and addiction‐like eating behavior in a feed‐forward fashion.^35^, ^36^ Evidence for the hypothesis suggesting that hypothalamic insulin resistance causes subsequent hyperphagia in PWS is seen in studies utilizing functional MRI scans, which show increased hedonic response to food in individuals with PWS.^36^, ^37^

Leptin uptake into the brain seems to follow a similar pattern to insulin, with low levels of leptin in the central nervous system relative to serum leptin, which has been deemed leptin resistance. It is thought that hypothalamic insulin resistance is partially responsible for leptin resistance.^38^ Lower levels of leptin in the central nervous system due to leptin resistance promote decreased energy expenditure and continued food consumption to make up for what the brain sees as an inadequate leptin level and starvation.

The strengths of this manuscript include the use of the nutritional phases in PWS to analyze the data; the expertize of the researchers who developed the widely accepted nutritional phases in PWS,^4^ the significant number of individuals in each nutritional phase; the two control groups (one normal weight and the other with early‐onset major obesity) that were matched for age; limiting participants to <13 years of age to minimize the effects of puberty, and the execution of all studies at a single site with the same investigators. The weaknesses of this manuscript include the inability to track the same individual through all the nutritional phases (due to the long span of years between phases), the small sample size for the oral glucose tolerance test (especially young children in nutritional phase 2a), the combined analysis of total body fat (DEXA) of boys and girls, and the exclusion of a significant number of participants with PWS and EMO from the study due to medications (e.g., diabetes medications) being taken which would have interfered with the study results.

## 5 CONCLUSIONS

Regulating insulin levels early in childhood before the onset of the early weight gain may be key in modulating the onset and severity of obesity and hyperphagia in individuals with PWS, as well as in other young children with non‐PWS early‐onset obesity. Preventing or reversing elevated insulin levels in PWS with pharmacological agents^39^, ^40^ and/or through diet restrictions such as a combined low carbohydrate, low glycemic‐load diet^41^, ^42^ may be a viable therapeutic strategy in combating obesity in children with PWS and others with early childhood obesity. In fact, one medication currently under investigation, Diazoxide Choline Controlled Release (DCCR), has shown promising results in clinical trials with individuals with PWS. Preliminary study data showed an improvement in hyperphagia, reduction in body fat, and increase in lean body mass.^43^ Also reported was a reduction in circulating lipids, leptin, insulin resistance, and waist circumference. Additional effects were improvements in aggressive and destructive behaviors.^43^

PWS is a powerful model system to understand obesity and appetite regulation given the remarkable changes individuals with PWS undergo with progression from anorexia and FTT in phase 1a to hyperphagia and lack of satiety in phase 3. Therefore, unraveling all the factors involved in the various nutritional phases in PWS will yield valuable insights into future treatments for individuals with PWS, as well as other types of childhood obesity. Future research endeavors will include the implementation of global metabolomics and lipidomics to evaluate the metabolic signature of each of the different nutritional phases in Prader‐Willi syndrome in the hope of further elucidating the mechanism(s) behind the hyperinsulinemia, hyperphagia and obesity that are hallmarks of this genetic condition.

## AUTHOR CONTRIBUTIONS

Drs. Driscoll and Kweh were responsible for the study design. Drs. Driscoll and Miller examined all the participants, determined the nutritional phases, and collected the study samples. Dr. Kweh and Mr. Sulsona performed the experiments, generated the data, and performed the literature searches. Dr. Kweh did the statistical analyses, generated the figures and tables, and wrote the first draft of the paper. All the authors had full access to the data, contributed to the discussion of the results, and edited the manuscript. Dr. Driscoll had primary responsibility for the final content.

## CONFLICT OF INTEREST STATEMENT

Frederick A. Kweh has received consulting fees from Soleno and Jennifer L. Miller has conducted clinical trials for Soleno. Daniel J. Driscoll and Carlos R Sulsona declare no conflict of interests.

## Supporting information

Supporting Information S1
