# Supplementary material for: Hyperinsulinemia is a probable trigger for weight gain and hyperphagia in individuals with Prader‐Willi syndrome
Source: Obes Sci Pract. 2023 Feb 17;9(4):383–94. doi: 10.1002/osp4.663 (PMC10399533; doi:10.1002/osp4.663)
Supplement: Supplementary file 1 — Supporting Information S1 [file OSP4-9-383-s001.pdf]

Hyperinsulinemia is a probable trigger for weight gain and hyperphagia in individuals with Prader-Willi syndrome

Frederick A. Kweh, Ph.D.[1,2]
Frederick.Kweh@icloud.com

Carlos R Sulsona, B.S.[1]
sulsonac@ufl.edu

Jennifer L. Miller, M.D., M.S.[1]
millejl@peds.ufl.edu

Daniel J. Driscoll, M.D., Ph.D.[1,3]
driscdj@peds.ufl.edu

[1] Department of Pediatrics, University of Florida, College of Medicine, Gainesville, FL, USA

[2]Current: Process and Analytical Development, Resilience Biotechnologies, Inc., Alachua, FL, USA

[3] Center for Epigenetics, University of Florida, College of Medicine, Gainesville, FL, USA

Supplementary Table 1: Tanner Stages for the 5-12 Year Participants

Group	Stage 1	Stage 2	Stage 3	Stage 4	Stage 5	n_{Total}
**PWS**	55% [11]	30% [6]	15% [3]	0% [0]	0% [0]	20
**EMO**	83% [15]	6% [1]	0% [0]	11% [2]	0% [0]	18
**SibC**	80% [36]	11% [5]	4% [2]	4% [2]	0% [0]	45

Note: Data expressed as percentage with sample size [n]. Population size denoted by 'N'. **N = 83**
